# Supplementary material for: Discovery and pharmacophoric characterization of chemokine network inhibitors using phage-display, saturation mutagenesis and computational modelling
Source: Nat Commun. 2023 Sep 16;14:5763. doi: 10.1038/s41467-023-41488-z (PMC10505172; doi:10.1038/s41467-023-41488-z)
Supplement: Supplementary file 4 — Source Data [file 41467_2023_41488_MOESM4_ESM.zip › SourceData/scripts/xoctet_20230808081637.tar!/xoctet/metadata/octet-biomolecular-binding-kinetics-application-note-4014-en-1--data.pdf]

Application Note

December 1, 2022

Keywords or phrases:
Octet®, Bio-Layer Interferometry, BLI, label-free, biosensor, biomolecular interactions analysis, affinity, kinetic characterization, binding kinetics, real-time kinetics, assay development and optimization

Biomolecular Binding Kinetics Assays on the Octet® BLI Platform

David Apiyo, Sartorius, Fremont, CA

Correspondence
Email: octet@sartorius.com

Abstract

Detecting and quantifying biomolecular interactions are critical in the discovery and selection of biotherapeutics leads. Octet® Bio-Layer Interferometry (BLI) systems implement a label-free analytical approach, which provides a fast and straightforward means to accurately determine binding specificity, affinity, and kinetics, which can be useful in every stage of biopharmaceutical development from early discovery to manufacturing. This application note introduces the family of Octet® BLI systems, the technology, capabilities, and advantages of label-free binding assessment in therapeutics development and life sciences research. It also provides guidance and best practices in biosensor assay development, optimization, data analysis, and reporting.

Contents

Introduction . . . 3
The Family of Octet® BLI Systems . . . 3
Dip and Read Biosensors . . . 4
Bio-Layer Interferometry . . . 6
Advantages of Label-free Analysis . . . 6
Binding Kinetics—Basic Principles . . . 7
Binding Kinetics Experiments . . . 7
Definition of Terms . . . 8
Relationship of R_{eq}, R_{max} and K_D . . . 9
Developing a Binding Kinetics Assay . . . 9
Choosing the Right Biosensor . . . 10
Streptavidin Biosensors for Kinetic Analysis . . . 10
Capture-Based Approach . . . 10
Assay Orientation . . . 10
Ligand Immobilization: Biotinylation of Protein for Immobilization onto Streptavidin Biosensors . . . 11
Immobilization Using Amine Reactive 2nd Generation (AR2G) Biosensors . . . 11
Optimizing Ligand Loading Density . . . 12
Assay Buffer . . . 13
Baseline Step . . . 13
Association Step . . . 14
Dissociation Step . . . 15
Non-specific Binding . . . 15
Biosensor Regeneration . . . 16
Running a Binding Kinetics Assay . . . 19
Analysis of Binding Kinetics Data . . . 20
Recognizing Non-ideal Binding Behavior . . . 20
Processing Parameters . . . 22
Analysis Window: Choosing a Curve Fit Model . . . 22
1:1 Binding Model . . . 23
Heterogeneous Ligand Model . . . 23
Mass Transport Model . . . 24
1:2 Bivalent Analyte . . . 24
Local Fitting: Full vs. Partial Fit . . . 25
Global Fit . . . 25
Evaluating Quality of the Fit . . . 26
Conclusion . . . 26

Introduction

Direct measurement of biomolecular interactions plays an important role in biotherapeutic drug discovery and development. Label-free analytical technologies such as the Octet® BLI platform from Sartorius provide a powerful means to obtain accurate information about rate of biomolecular complex formation and complex stability, key components of a drug-target interaction. The affinity of an interaction directly affects the dose at which a biopharmaceutical is effective, and understanding mechanism of binding has implications in efficacy and desirability of a therapeutic candidate. Real-time data on specificity, affinity and kinetics of binding interactions can benefit every stage of biopharmaceutical development, from early discovery to the manufacturing process (Figure 1).

Efficient selection of therapeutic candidates early in the drug development process can save both time and resources and prevent late-stage failures. For example, high–throughput off-rate ranking enables selection of highest affinity clones in primary screening assays in crude samples. Coupling real-time kinetic analysis with lead optimization techniques such as affinity maturation can provide valuable information on affinity and binding mechanisms of lead candidates during development. Accordingly, downstream processes require detailed characterization of specificity, selectivity, stability and binding rate analyses under various growth or buffer conditions to facilitate informed process decisions.

While the value of kinetic analysis of molecular interactions using label-free technology is clear, there are many practical considerations involved in producing consistent, high quality kinetic binding profiles from biological samples. Here we introduce large molecule kinetic analysis on the Sartorius Octet® platforms using Bio-Layer Interferometry, and discuss options, techniques and considerations for developing and performing successful kinetic characterization assays and interpreting results.

The Family of Octet® BLI Systems

Sartorius offers a variety of multi-functional instrument platforms based on Bio-Layer Interferometry (BLI), a label-free technology that measures molecular interactions in real time for the purpose of detection, quantitation and kinetic analysis. Octet® BLI systems utilize a standard microplate format which enables high-throughput, automated binding analysis in 96-well and 384-well microplates, with minimal instrument maintenance and flexible assay design.

Several instrument options are available depending on sensitivity and throughput requirements. The Octet® R2, R8, RH16 and RH96 BLI systems offer the best performance for quantitation and kinetic analysis, with the broadest dynamic range and highest sensitivity. These systems have the ability to measure fast binding interactions and protein-small molecule binding interactions, with sensitivity down to a molecular weight of 150 Daltons. The Octet® RH16 and RH96 BLI systems each have two plate positions and analyze up to 16 samples and 96 samples in parallel in 96- or 384-well microplates. These systems are capable of higher throughput for faster time to results in the same application areas covered by the Octet® R2 BLI system that can run two samples, and the Octet® R8 and R4 BLI systems which can run up to eight samples in parallel in 96-well microplates. The Octet® RH16 and RH96 BLI systems also have reduced sample

Figure 1: *Kinetic Applications of Octet® BLI Systems in the Biopharmaceutical Research and Development Process.*

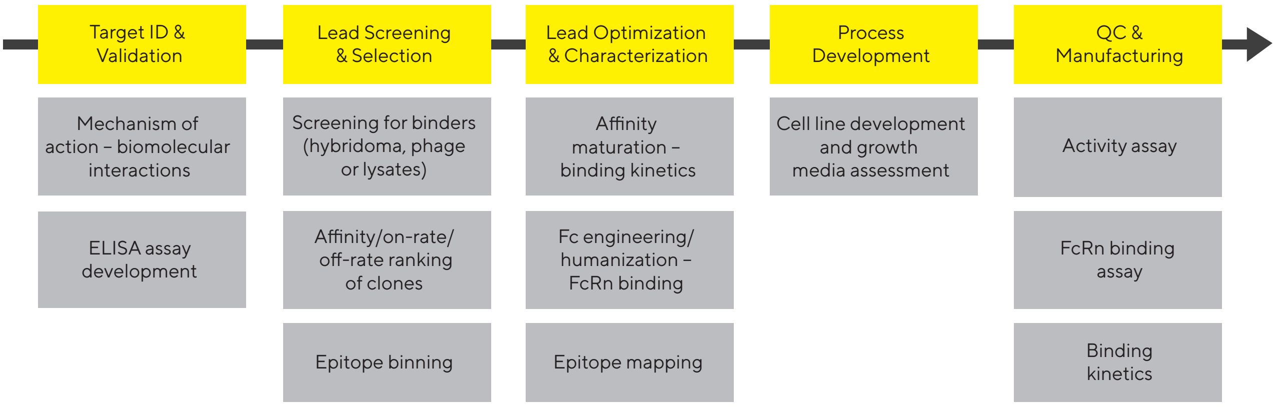

volume requirements, as low as 40 µL/sample when 384-well tilted bottom plates are used. Table 1 describes each of the Octet® BLI systems and dynamic ranges for kinetic assays.

Dip and Read Biosensors

Biosensors used with the Octet® BLI system are single-use and disposable. The tip of each fiber-optic biosensor is coated with a proprietary biocompatible matrix that minimizes non-specific binding to the surface. This matrix is coated with one of a wide selection of biosensor chemistries available for highly specific binding of target molecules in a sample. The biosensor chemistries available for kinetics applications are listed in Table 2.

Octet® BLI platform combined with the Dip and Read format of the biosensors offer several advantages over other label-free technologies. Sample volume is one consideration. The microplate format enables highly parallel processing in small volumes of as small as 40 µL. Samples are not consumed or destroyed by analysis, and can be recovered once the assay is complete. Because groups of biosensors are moved in parallel between samples held in microplates, assay time is significantly reduced relative to flow-based SPR systems. Without microfluidics, contamination of instrument components is not an issue and there is no risk of clogging when using crude, unpurified samples. Procedures such as instrument maintenance, desorption protocols, degassing of buffers, unclogging and flushing of tubing are also eliminated. Cost however the Octet® platform's single-use biosensors are disposable and cost-effective, offering an advantage over SPR and other platforms where consumables are much more costly and difficult to manufacture. Although biosensors can be disposed of after a single use, regeneration is possible in many cases, resulting in even further reduced cost per assay.

Table 1: *Selected Performance Specifications for Octet® BLI Systems.*

	**Octet® R2 BLI System**	**Octet® R4 BLI System**	**Octet® R8 BLI System**	**Octet® RH16 BLI System**	**Octet® RH96 BLI System**
**Performance**					
Maximum simultaneous reads	2	4	8	16	96
Molecular weight	>150 Da	>150 Da	>150 Da	>150 Da	>150 Da
On-rate (k_a) range ($M^{-1}s^{-1}$)	$10^1 – 10^7$	$10^1 – 10^7$	$10^1 – 10^7$	$10^1 – 10^7$	$10^1 – 10^7$
Off-rate (k_d) range (s^{-1})	$10^{-6} – 10^{-1}$	$10^{-6} – 10^{-1}$	$10^{-6} – 10^{-1}$	$10^{-6} – 10^{-1}$	$10^{-6} – 10^{-1}$
Affinity (K_D) range	1 mM – 10 pM	1 mM – 10 pM	1 mM – 10 pM	1 mM – 10 pM	1 mM – 10 pM
Evaporation control	No	Yes	Yes	No	No
Minimum sample volume	200 µL	200 µL	200 µL	40 µL*	40 µL*
Acquisition rate (Hz)	2, 5, 10	2, 5, 10	2, 5, 10	2, 5, 10	0.3, 0.6, 2, 5, 10
**Specifications**					
Number of spectrometers	2	4	8	16	16
Temperature Control	Ambient +4 – 40°C	15 – 40°C	15 – 40°C	Ambient +4 – 40°C	Ambient +4 – 40°C
Microplate positions	1 (96-well)	1 (96-well)	1 (96-well)	2 (96- or 384-well)	2 (96- or 384-well)
Integration with automation	No	No	No	Yes	Yes
**Dimensions**					
Size H × W × D (cm)	49 × 56 × 46	49 × 56 × 46	49 × 56 × 46	77 × 80 × 80	77 × 80 × 80
Weight (kg)	32.7	32.7	32.7	68.2	90.7

* In a 384-well tilted-bottom microplate (Sartorius, part no. 18-5080)
** In a 96-well half-area microplate (VWR, part no. 82050-044)

Table 2: *Biosensors Available for Kinetic Assays on the Octet® BLI Systems.*

Biosensor	Description
Anti-hIgG Fc Capture (AHC)	Immobilization of human IgG or other human Fc-containing proteins by binding to the human Fc region. Kinetic applications include protein and antibody kinetic screening, affinity characterization (k_a, k_d, K_D), epitope binning, and hit validation.
Anti-hIgG Fc Capture for Quantitation (AHQ)	Quantitation measurements of human IgG's or human Fc-fusion proteins
Anti-Mouse Fc Capture (AMC)	Binds the Fc portion of IgG1, IgG2a, IgG2b for capture-based immobilization. Applications include kinetic analysis of antibody-antigen interactions (k_a, k_d, K_D) and off-rate screening. IgG3 should be evaluated on a case-by-case basis.
Anti-Mouse Fc Capture for quantitation (AMQ)	Quantitation measurements of mouse IgG's or mouse F(ab')2
Anti-Human Fab-CH1 2nd generation (FAB2G) (FAB2)	Binds specifically to the CH1 domain of human IgG, allowing quantitation and kinetic characterization of human Fab, F(ab')2, and IgGs with no recognition towards free light chains.
Protein A (ProA)	Protein A coated biosensor for capture and quantiation of IgG's of various species.
Protein G (ProG)	Protein G coated biosensor for capture and quantitation of IgG's of various species.
Portein L (ProL)	Protein L coated biosensor for capture and quantitation of IgG's of various species
Streptavidin (SA)	Streptavidin-coated biosensor with high binding capacity. Immobilizes biotinylated antibodies, proteins, and nucleic acids to form a stable surface. Kinetic applications include protein and antibody kinetic screening, affinity characterization (k_a, k_d, K_D), epitope binning, and hit validation.
High Precision Streptavidin (SAX)	Streptavidin-coated biosensor for Immobilizing biotinylated molecules for high precision quantitation and kinetic measurements
High Precision Streptavidin 2.0 (SAX2)	Streptavidin-coated biosensor with reproducible biotin-ligand loading from one lot to another. QC tested to limit biotinylated ligand loading variance, providing confident detection of variance from samples. Suited for product release QC, activity assays in manufacturing and other highly precise quantitation and kinetic applications.
Super Streptavidin (SSA)	Streptavidin-coated biosensor with a very high density of biotin binding sites. Immobilizes biotinylated proteins, peptides, nucleic acids and small molecules to form a stable surface. Kinetic applications include antibody, protein, peptide, and small molecule kinetic screening, affinity characterization (k_a, k_d, K_D), epitope binning, and hit validation.
Anti-GST (GST)	High affinity anti-GST antibody enables direct and rapid quantitation of GST-tagged biomolecules and easy and reliable capture of GST-tagged biomolecules for rapid kinetic analysis.
Anti-Penta HIS (HIS1K)	Capture of His-tagged proteins for kinetic analysis with target analytes. Quantitation of His-tagged proteins in buffer, media or diluted lysate. The sensor is pre-coated with Penta-His antibody from Qiagen.
Anti-HIS (HIS2)	Quantitation of HIS-tagged proteins in crude matrices or buffer or column eluent. The sensor is pre-coated with anti-His Ab from MBS.
Ni-NTA (Ni-NTA)	Nickel charged tris-NTA with strong binding to HIS-tagged recombinant proteins for kinetic measurements and quantitation.
Amine Reactive Second Generation (AR2G)	Second-generation carboxylate-functionalized surface allows covalent coupling of proteins via EDC/s-NHS mediated amide bond formation. The second-generation surface provides increased loading density, more robust loading conditions and decreased non-specific binding compared to the original AR Biosensor for most proteins. Kinetic applications include protein and antibody kinetic screening, affinity characterization (k_a, k_d, K_D), and hit validation. Requires AR2G Assay Kit (part no. 18-5095).
Aminopropylsilane (APS)	Adsorption of proteins and membrane fractions through hydrophobic moieties. Kinetic applications include adsorption of proteins and membrane fractions through hydrophobic moieties for kinetic screening and affinity characterization (k_a, k_d, K_D).

Bio-Layer Interferometry

BLI is an optical analytical technique that measures interference patterns between waves of light. White light is directed down the fiber-optic biosensor towards two interfaces separated by a thin layer at the tip of the fiber: a biocompatible layer on the surface of the tip, and an internal reference layer (Figure 2). Light reflects from each of the two layers, and the reflected beams interfere constructively or destructively at different wavelengths in the spectrum. This interference pattern is detected at the CCD array detector.

Figure 2: *A Dip and Read Biosensor.*

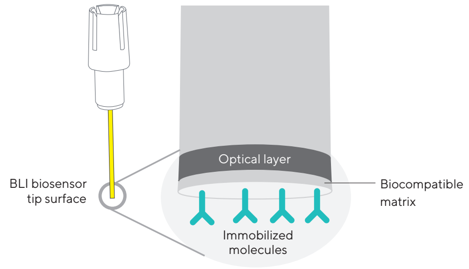

Note. Illustrating the two optical interfaces at the biosensor tip the internal reference layer (optical layer) and the surface biocompatible matrix on which ligand molecules are immobilized.

When the tip of a biosensor is dipped into a sample in a 96-well microplate, target molecules bind to the 2-dimensional coated surface. This binding forms a molecular layer that increases in thickness as more target molecules bind to the surface. As the thickness at the tip increases, the effective distance between the two reflective layers increases, creating a shift in the interference pattern of the reflected light (Figure 3). The spectral pattern of the reflected light therefore changes as a function of the optical thickness of the molecular layer, *i.e.*, the number of molecules bound to the biosensor surface. This spectral shift is monitored at the detector, and reported on a sensorgram as a change in wavelength (nm shift). Monitoring the interference pattern in real time provides kinetic data on molecular interactions.

Figure 3: *BLI Optical Analytical Technique.*

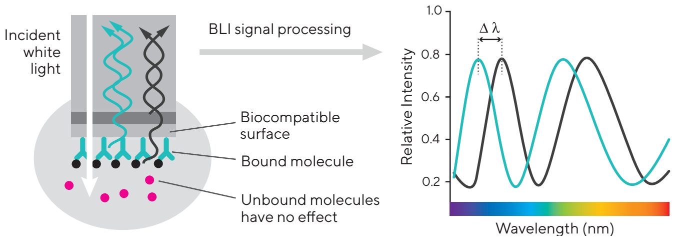

Note. BLI is an optical analytical technique that analyzes the interference pattern of white light reflected from two surfaces. Changes in the number of molecules bound to the biosensor causes a shift in the interference pattern that is measured in real time.

Advantages of Label-free Analysis

Kinetic characterization with the Octet® BLI system can complement or replace label-dependent methods such as ELISA or TR-FRET, which are less efficient and informative for analyzing molecular interactions. Generating labeled biomolecules not only consumes time and material but can lead to undesirable results such as altering protein activity or steric blocking of binding sites. Real-time, label-free analysis provides rapid, sensitive and accurate measurement of kinetics, affinity and activity of complex formation without artifacts or issues associated with traditional techniques.

In contrast to standard endpoint assays, it is possible to derive detailed information about the mechanism of interaction between molecules using Octet® BLI systems. Equilibrium binding assays can determine the affinity constant, or K_D, but cannot provide information about binding rates. IC_{50} values can approximate K_D under optimal conditions, however different combinations of on-rate and off-rate can yield the same affinity constant even when the actual binding profile may differ greatly. Figure 4 illustrates sensorgram traces from two binding pairs with the same affinity constant, or K_D, however the binding profile for interaction A exhibits a much slower on-rate and off-rate than the profile for interaction B. This example demonstrates how real-time binding assays provide a more complete understanding of an interaction by providing both affinity and binding rate data.

Real-time kinetic analysis can also shed light on whether binding exhibits simple 1:1 stoichiometry or whether a more complex interaction is taking place. By obtaining information on the nature of binding interactions, proteins that have high affinity but exhibit non-optimal binding behavior can be quickly eliminated as lead drug candidates early on in the process. In addition, high sensitivity measurement of binding affinities up to the millimolar range are possible, whereas techniques such as ELISA or immunoprecipitation assays require washing steps where weaker binders can be lost.

Figure 4: *Sensorgram Traces.*

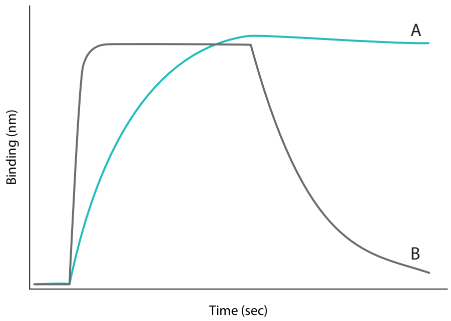

Note. Sensorgram traces from two interactions having similar affinity constants but different binding profiles.

Binding Kinetics—Basic Principles

Kinetic analysis is used to determine affinity of an interaction and measure association and dissociation rate constants for reversible, non-covalent binding. Non-covalent binding is typically comprised of a combination of ionic/electrostatic interactions, hydrogen bonds, van der Waals forces, and hydrophobic effects. The specific recognition and binding of biological molecules by antibodies and other proteins is fundamental to many processes in biology.

Binding Kinetics Experiments

In biosensor analysis, one interactant is immobilized on the surface of the biosensor (ligand) and the other remains in solution (analyte). A typical binding kinetics experiment using Dip and Read biosensors on the Octet® platform is illustrated in the diagram in Figure 5A. The assay begins with an initial baseline or equilibration step using assay buffer. Next, a ligand molecule such as an antibody is immobilized on the surface of the biosensor (loading), either by direct immobilization or capture-based method. After ligand immobilization, biosensors are dipped into buffer solution for a baseline step to assess assay drift and determine loading level of ligand. After the baseline step, biosensors are dipped into a solution containing the ligand's binding partner, the analyte (association). In this step, the binding interaction of the analyte to the immobilized ligand is measured. Following analyte association, the biosensor is dipped into buffer solution without analyte, and the bound analyte is allowed to come off the ligand (dissociation). Several concentrations of analyte are run in parallel (up to eight measurements simultaneously on the Octet® R2 and R8 BLI systems, and up to 16 measurements simultaneously on the Octet® RH16 BLI system). The series of assay steps is then repeated on new or regenerated biosensors for each analyte being tested. Each binding response is measured and reported in real time on a sensorgram trace. A typical sensorgram for a single sample is illustrated in Figure 5B.

Figure 5: *A Typical Binding Kinetics Experiment Using Streptavidin Biosensors.*

A.

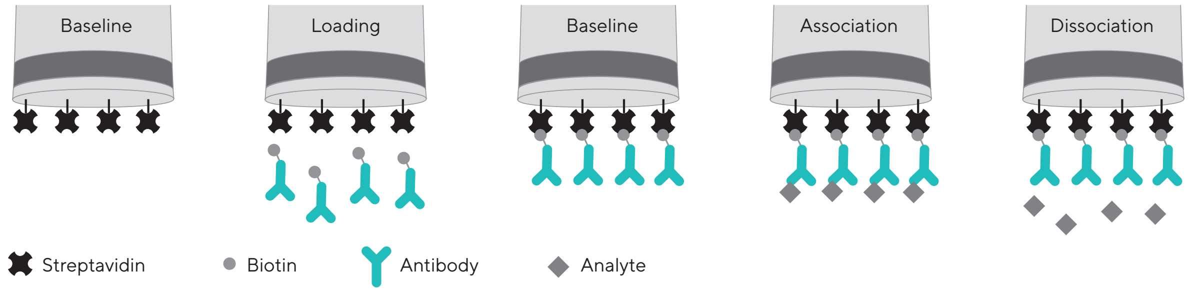

B.

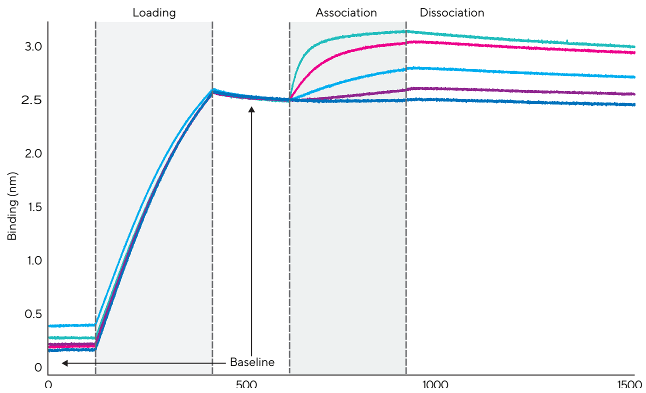

Note. A) After an initial baseline step in buffer, biosensors are dipped into solution with biotinylated ligand. In this example, ligand molecule is an antibody. A second baseline step is performed followed by association and dissociation of analyte molecule in solution. B) A raw data sensorgram showing real-time data acquisition for each step of a kinetic assay.

Definition of Terms

Kinetics refers to how fast an interaction occurs. Association measures how fast one molecule binds to another, and dissociation measures how fast a complex falls apart. After a certain period of time, equilibrium will be established and complexes will associate at the same rate as they dissociate, so the number of bound and unbound molecules remains constant. Affinity measures how strong the complex is, specifically, how much complex is formed when binding reaches equilibrium. The simplest model used to describe this interaction between two biomolecules is represented by the equation below:

$$A + B \underset{k_d}{\overset{k_a}{\rightleftharpoons}} AB$$

where A represents the ligand molecule immobilized on the surface of the biosensor, and B is the analyte in solution. This binding model assumes a simple 1:1 interaction, where one ligand molecule interacts with one analyte molecule, and binding is independent and of equal strength for all binding sites. Complex formation in this case follows pseudo-first-order kinetics. The rate of complex formation (AB) is dictated by an association rate constant and the concentrations of unbound ligand and analyte. The equation used to fit the association is an integration of a differential equation showing that the rate of association is a function of the decreasing concentration of unbound ligand molecules as analyte binding occurs:

$$Y=Y_0+A(1-e^{-k_{obs}*t})$$

where Y = level of binding, Y_0= binding at start of association, A is an asymptote and t = time. k_{obs} is the observed rate constant. At the same time AB complex is forming, it also decays back to A and B. The term k_{obs} reflects the overall rate of the combined association and dissociation of the two binding partners.

When biosensors are dipped into buffer solution that is free of analyte, the complex begins to decay at a rate that is a function of the dissociation rate constant and the concentration of ligand-analyte complex. The equation used to fit dissociation is:

$$Y=Y_0+Ae^{-k_d*t}$$

where Y_0 is binding at start of dissociation, and k_d is the dissociation rate constant. This equation reflects the decline in the dissociation rate over time as the concentration of bound complexes on the surface decreases. The term k_d measures the stability of the complex, or the fraction of complexes that decay per second, and is expressed in units of sec^{-1}. By solving the above equations for k_{obs} and k_d, the association rate constant ka can then be calculated with the equation:

$$k_a= \frac{k_{obs} - k_d}{[Analyte]}$$

The association rate constant, ka, represents the number of AB complexes formed per second in a 1 molar solution of A and B. The term ka is expressed in $M^{-1}sec^{-1}$.

K_D is the affinity constant, or equilibrium dissociation constant, which measures how tightly the ligand binds to its analyte. It represents the ratio of the on-rate to the off-rate and can be calculated using k_a and k_d:

$$K_D= \frac{[A]\cdot[B]}{[AB]} = \frac{k_d}{k_a}$$

K_D is expressed in molar units (M). The K_D corresponds to the concentration of analyte at which 50% of ligand binding sites are occupied at equilibrium, or the concentration at which the number of ligand molecules with analyte bound equals the number of ligand molecules without analyte bound. There is an inverse relationship between K_D and affinity—a smaller affinity constant indicates a tighter interaction, or greater affinity of analyte to ligand.

In order to calculate K_D and k_a, the concentration of the analyte must be known. k_d, however, is concentration independent. The larger the k_d, the faster the dissociation is occurring. Table 3 shows the relationship between k_d values and half-life of bound complex.

Because k_d can be determined without knowing how much analyte is present, it is useful for screening applications such as ranking sets of unpurified binders in complex matrices.

Table 3: *Relationship Between Off-rate (k_d) and Half-life of Bound Ligand-analyte Complex.*

k_d	% Dissociation per Second	Time to 50% Dissociation
1	100	0.69 seconds
0.1	10	6.93 seconds
1×10^{-2}	1	69.3 seconds
1×10^{-3}	0.1	11.55 minutes
1×10^{-4}	0.01	1.93 hours
1×10^{-5}	0.001	19.25 hours
1×10^{-6}	0.0001	8 days

Relationship of R_{eq}, R_{max} and K_D

If the analyte association phase is left to run long enough, eventually the binding interaction will reach a point where rates of association and dissociation are equal. At this point the curve flattens out and has reached the equilibrium binding signal, or R_{eq}. Since there is a fixed amount of ligand on the surface of the biosensor, there is a maximum amount of analyte that can be bound. When the highest possible analyte binding is reached, it is considered the maximum binding signal, or R_{max}. The value of R_{eq} relative to R_{max} depends on the analyte concentration relative to the K_D. For example, if the analyte concentration is equal to the K_D, R_{eq} will occur at 50% of R_{max}. R_{max} will generally be reached if the analyte concentration is greater than 10X K_D (Figure 6).

Figure 6: *Diagram Showing the Relationship Between R_{eq}, R_{max}, and K_D in an Ideal Interaction.*

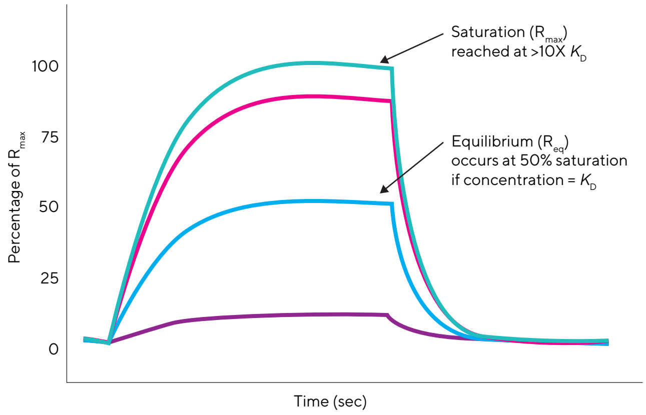

Note. The top data trace demonstrates saturation of the assay which typically occurs at greater than 10X K_D. The equilibrium portions of data traces will begin to stack together even when analyte concentration is increased, because all of the available ligand molecules have been bound. K_D is the concentration where equilibrium is reached at 50% saturation.

Developing a Binding Kinetics Assay

Setting up a kinetic assay using the Octet® platform is simple and straightforward. However, appropriate choice of biosensor chemistry and use of optimized experimental conditions are critical to determining accurate affinity and kinetic constants. Proper assay technique and optimization as well as use of high quality, active reagents are key to obtaining accurate data from a binding kinetics experiment. Use of unstable or inactive proteins, improperly characterized reagents, or inappropriate buffer conditions will negatively impact results. Performing interaction analysis on an active and stable ligand surface facilitates generation of robust data. Below, we describe considerations for developing kinetic assays in various formats, with suggestions for designing an effective assay, optimizing each step, and minimizing artifacts arising from non-specific binding.

Choosing the Right Biosensor

Analysis of biomolecular interactions starts with immobilization of a ligand onto the surface of the biosensor. Biosensors come ready to use with standard binding agents such as streptavidin and amine-reactive groups, allowing for irreversible attachment of proteins to essentially create custom biosensor surfaces. Alternatively, capture agents such as anti-mouse IgG Fc capture or Ni-NTA provide a means for highly specific capture of antibodies or recombinant proteins even from unpurified samples. The most important consideration for biosensor selection is choosing a format that best maintains structure and activity of the immobilized ligand.

Direct immobilization of a target protein to a biosensor can be accomplished by covalent bond to free lysine residues via an Amine Reactive biosensor (AR2G) or via biotin interaction with Streptavidin Biosensor. Direct immobilization results in stable, non-reversible coupling of a molecule to the biosensor with minimal dissociation from the biosensor. This method is compatible with most proteins, enables creation of a 'custom' biosensor surface with virtually any protein, and is typically regenerable to the level of the immobilized ligand. Some constraints apply to this method. Direct immobilization requires purified protein and creation of a covalent bond, either directly to the surface or to biotin. There is potential for loss of protein activity due to covalent bonding or to steric hindrance, especially when the biosensor surface is overly saturated. With direct coupling using Amine Reactive Biosensors, the surface is activated and then the protein exposed to the activated surface at low pH (typically pH 5.5). It is difficult to control this type of reaction and ensure the protein is not overly constrained by the surface. If too many lysines are linked to the surface, or free lysines near the analyte binding site are linked, the protein may lose activity.

Streptavidin Biosensors for Kinetic Analysis

Streptavidin Biosensors have been developed for the immobilization of biotinylated ligands. The interaction between streptavidin and biotin is non-covalent, but is rapid, stable and essentially irreversible. Use of Streptavidin Biosensors as an alternative to amine covalent coupling offers some significant advantages. The biotin-streptavidin coupling method creates a stable bond to the surface on the biosensor similar to covalent coupling, yet requires minimal optimization. Biotinylation is simple to perform, gentle on proteins and performed at neutral pH. The reaction is easily controlled by regulating the number of biotin molecules added to the reaction per target protein, thus preventing activity loss due to over-modification. Long chain linkers should be incorporated into a biotin tag to minimize steric effects, especially when immobilizing smaller molecules such as peptides. A biotinylated ligand can be prepared in batches and stored for use in multiple experiments, whereas the amine reactive process involves multiple steps with reagents that must be prepared fresh with each immobilization.

Capture-Based Approach

Site-directed or capture-based biosensors can be used to maximize activity of the ligand on the surface. Capture biosensors are pre-immobilized with a high affinity capture antibody or protein which binds to the protein ligand via a known motif or tag, enabling favorable orientation on the surface and improved homogeneity. For example, anti-human and anti-mouse IgG Fc capture surfaces bind an antibody ligand via the Fc region, orienting the captured antibody so that the Fv region is readily available for analyte binding. Several oriented-capture biosensors are available for use on the Octet® platform (see Table 2). Because of the high specificity of these interactions, ligand protein can be captured directly from crude samples such as culture media without need for purification. Minimal assay optimization is required with capture-based biosensors, and they are typically regenerable to the level of the original capture surface. Several of the capture biosensors are antibody-based, and there may be some level of dissociation of the captured ligand observed. This background dissociation must be subtracted out using a no-analyte reference sample in order to calculate accurate kinetic constants (see Data Analysis section for information on reference subtraction). For the assay to be valid, as a general rule the dissociation rate of the analyte must be at least five times slower than the dissociation rate of the ligand from the biosensor.

Assay Orientation

Some consideration must be taken in determining which member of a binding pair to immobilize and which should remain in solution. Protein stability, size, and valency are primary concerns when choosing orientation. More sensitive proteins may not tolerate the relatively harsh conditions imposed by immobilization on amine reactive surfaces. In this case, immobilize the less sensitive binder of the pair. Alternatively, a different biosensor such as Streptavidin may be chosen. Since smaller molecules produce smaller signals, instrument sensitivity must also be considered when deciding which molecule to immobilize (see The Family of Octet® BLI Systems section). A smaller molecule such as a peptide can be immobilized onto the biosensor, with the larger binder in solution, to increase assay signal. If this

strategy is chosen, adding a linker to the captured molecule before immobilization can prevent steric hindrance and make the binding site more available to its analyte partner. While the affinity of a molecule to its binding partner is defined as the strength of the non-covalent association between one ligand binding site with one analyte binding site, the avidity of a molecule is determined by the total strength of all the binding associations possible between the two molecules. For example, an antibody is bivalent, and can bind more than one antigen epitope. Avidity is an important consideration in kinetics experiments as it can affect the overall calculation of K_D. Bivalent molecules such as antibodies should be immobilized on the surface of the biosensor whenever possible to avoid avidity effects. A bivalent antibody in solution can potentially interact with two antigen molecules on the surface (Figure 7).

Avidity effects can create non-ideal binding profiles and result in an artificially high affinity measurement. Regardless of orientation or assay format, proper assay development is a necessity for obtaining reliable kinetic data with any system.

Figure 7: *Avidity Effects in a Kinetic Assay.*

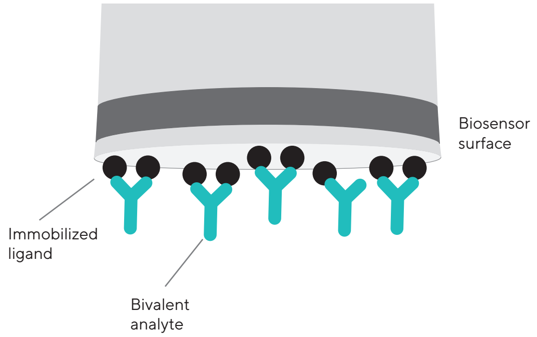

Note. A bivalent analyte in solution that binds to immobilized ligand has the potential to bind to two ligand molecules at once. This can result in artificially low apparent off rates. These effects can be prevented by reversing the assay format or by lowering the ligand density on the biosensor.

Ligand Immobilization: Biotinylation of Protein for Immobilization onto Streptavidin Biosensors

To immobilize a ligand onto a Streptavidin Biosensor, it must first be biotinylated. *In vivo* site-specific biotinylation methods that place one streptavidin binding site at a carefully chosen location on the ligand are recommended. However, when opting to perform biotinylation in the lab, the following recommendations should be followed. Proteins to be biotinylated must be purified, carrier protein-free and in a buffer that does not contain primary amines, such as Tris or glycine. A variety of biotinylation reagents targeting different functional groups are available commercially that allow for simple and efficient attachment of biotin to antibodies, proteins or peptides. The most commonly used are NHS-esters of biotin that target primary amines such as the amine group of free lysine residues in a protein or peptide. Spacer arms, or linkers, of different lengths are available, and are necessary to reduce steric hindrances and for efficient capture of biotinylated molecules. We recommend reagents such as EZ-Link NHS-PEG4-Biotin (Thermo Scientific, part no. 21329), NHS-PEG12-Biotin (Thermo Scientific, part no. 21338) or Sulfo-NHS-LC-LC-Biotin (Thermo Scientific, part no. 21338). Peptides can be synthesized with a biotin with a long linker incorporated. An optimal ratio of biotin reagent:ligand is a 1:1 molar coupling ratio (MCR). Over-biotinylation does not improve biosensor loading levels and has the potential to reduce protein activity due to increased likelihood of biotin incorporation into binding sites. A higher MCR may be necessary if the stock protein is at a concentration of less than 500 µg/mL and in this case a 3:1 or 5:1 ratio may be used. After biotinylation, excess unincorporated biotin should be removed. Unincorporated biotin can compete for streptavidin binding sites and reduce labeled ligand immobilizations to biosensors. Gel filtration spin columns that are used for buffer exchange and desalting are widely used for removing unincorporated biotin for its ability to separate molecules based on the molecular weights. Alternatively, dialysis into PBS buffer with an appropriate molecular weight cutoff membrane or cartridge can be used for gentle buffer exchange of more sensitive proteins (use 100 kDa MW cutoff for antibodies). Once the protein has been biotinylated and desalted into neutral pH buffer, a ligand loading optimization experiment can be performed to determine optimal loading concentration. For a detailed protocol for biotinylation of protein ligands for use on Streptavidin Biosensors, refer to Sartorius Technical Note 28, Biotinylation of Protein for Immobilization onto Streptavidin Biosensors.

Immobilization Using Amine Reactive 2nd Generation (AR2G) Biosensors

Immobilization of proteins on AR2G Biosensors is achieved through standard EDC-catalyzed amide bond formation to create a covalent bond between a reactive amine on the protein and the carboxy-terminated biosensor surface. The immobilization is done in a series of steps: 1) carboxylic acid groups on the surface are first activated by reaction with EDC (1-Ethyl-3-[3-dimethylaminopropyl] carbodiimide hydrochloride) and sulfo-NHS (N-hydroxysulfosuccinimide) to generate highly reactive NHS esters. 2) The esters rapidly react with the primary amines of the ligand biomolecule in

the coupling step to form highly stable amide bonds. 3) Excess sulfo-NHS esters are quenched using ethanolamine (Figure 8).

Pre-concentration of the ligand at the biosensor surface is critical for efficient immobilization and optimal signal to noise ratio in a kinetics assay. In pre-concentration, the ligand is concentrated at the surface by electrostatic attraction prior to covalent coupling. For these electrostatic interactions to occur, the pH of the coupling buffer must be higher than the pKa of the biosensor surface but slightly lower than the isoelectric point of the ligand. The buffer must also have low ionic strength. For many proteins, diluting 10 mM acetate buffer at pH 4.5–5 works well, however an optimization step, or pH scouting, is recommended to identify the optimal buffer in which to perform the immobilization. For scouting, choose three to four buffers differing by 0.5 or 1 pH unit, for example 10 mM sodium acetate pH 4, 5, and 6. Perform the immobilization protocol and the analyte association step using a high concentration of analyte (10–20X K_D). Select the buffer condition that produces the highest signal in the analyte association step for the assay, and then optimize for loading concentration. For detailed protocol on AR2G Biosensor immobilization, refer to Sartorius Technical Note 26, Dip and Read Amine Reactive Second-Generation (AR2G) Biosensors.

Optimizing Ligand Loading Density

Loading the optimal density of immobilized ligand on the surface of a Streptavidin or Amine Reactive Biosensor is critical to obtaining quality kinetic data. Simply loading as much ligand as possible to maximize signal is not recommended. An excess of ligand bound to the biosensor can lead to data artifacts due to crowding, steric hindrance and possible aggregation on the surface. Over-saturation of the biosensor may also promote weaker non-specific interactions at higher analyte concentrations, or analyte 'walking' or 'rebinding' effects at lower analyte concentrations. These artifacts may significantly impact observed binding kinetics. If not enough ligand is immobilized, however, the signal in the analyte association step may be too low to detect. When performing the loading step in a kinetic assay, slow loading for longer time is preferable to rapid ligand immobilization. Ideally, the binding curve in the loading step will show a gradual increase in signal and should not be allowed to reach saturation. A steep, fast initial increase in signal may lead to uneven loading and assay artifacts. A typical immobilization concentration for a ligand molecule is 50–100 nM. If the ligand concentration is low, *e.g.* below 50 nM, a longer loading time may be required for sufficient immobilization signal. Overnight incubation in ligand solution may also be performed at 4°C. Overnight incubation can greatly improve results in cases where capture biosensors are being used to capture a ligand molecule from a dilute supernatant or cell culture sample.

Determining the ideal concentration of ligand to immobilize requires experimentation. An assay development step in which the ligand is titrated on the biosensors is recommended. The microplate format used on the Octet® platform allows for rapid testing of several experimental parameters at once, minimizing time spent on assay development. To perform a loading optimization experiment, several concentrations of ligand are loaded onto the biosensor. An association step is performed for each ligand concentration using a high concentration of analyte (10–20X K_D). A zero-ligand biosensor should also be run as a control for determining whether the analyte binds non-specifically to the biosensor.

Figure 8: *Workflow for a Kinetic Assay Using AR2G Biosensors.*

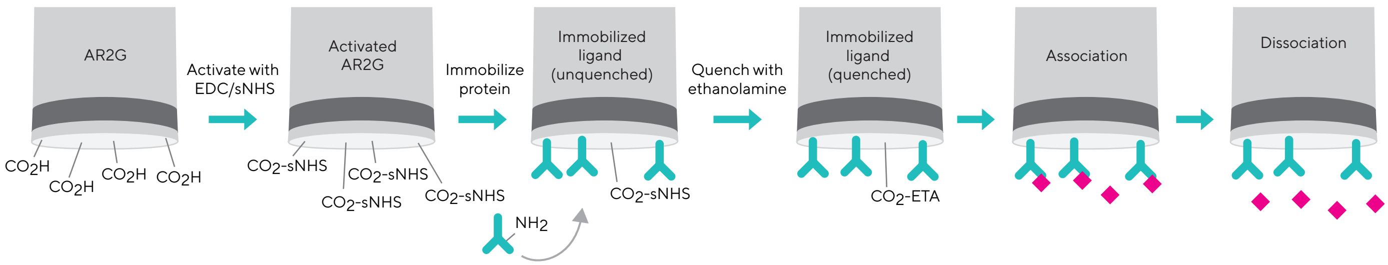

Note. Ligand molecules are covalently attached to the activated amine-reactive surface. After activation, immobilization and quenching, the kinetics of association and dissociation between the immobilized ligand and analyte are measured.

The loading concentration to select for an assay should be the lowest concentration of immobilized ligand that yields an acceptable signal in the analyte association step. Figure 9A shows the raw data trace for a ligand loading optimization experiment. In the loading step, higher concentrations of ligand quickly saturate the binding sites, as evidenced by the binding curve leveling off in the 100 µg/mL sample, while the lowest concentrations do not reach saturation. When the data are processed so that the analyte association step is aligned to the baseline (Figure 9B), the relative signal of the analyte binding at each corresponding ligand concentration can be clearly observed. At 10 µg/mL (67 nM), we observe desired loading curve characteristics (significant loading signal with fairly slow initial binding that does not go to saturation) and a high signal in the association step. This concentration should be selected as the optimal loading concentration for this example.

Assay Buffer

The Dip and Read format of the Octet® BLI system allows for greater flexibility in selecting an assay buffer than alternative platforms such as SPR. Because biosensors are based on BLI technology, only molecules binding to or dissociating from the surface of the biosensor can shift the interference pattern of the reflected light and generate a response. Unbound molecules or changes in refractive index of the surrounding solution do not affect the interference pattern, enabling measurements in crude or complex samples such as cell lysates or culture supernatants, and in solutions containing high refractive index components such as glycerol or DMSO.

Though the Octet® BLI system offers a great deal of flexibility in choice of assay and sample matrix, be sure to select an assay buffer that is appropriate for the experimental system, and use the same solution throughout the assay. For example, if the analyte is in culture media, make analyte dilutions in the same media and use this media for the baseline and dissociation steps as well. For kinetic assays using purified samples we recommend using Sartorius' Kinetics Buffer as a sample buffer, which is available as a 10X solution. This buffer contains the blocking agent, bovine serum albumin (BSA), and surfactant (Tween-20) to inhibit non-specific binding to surfaces and other proteins.

Baseline Step

A baseline step must be performed prior to analyte association to remove unbound ligand from the biosensor, and to assess drift caused by non-specific binding, aggregation or buffer effects. A baseline is run in buffer (or media) that must match the buffer to be used for the association and dissociation steps. In many cases this solution differs from

Figure 9: *Sensorgram Data Illustrating Ligand Loading Optimization.*

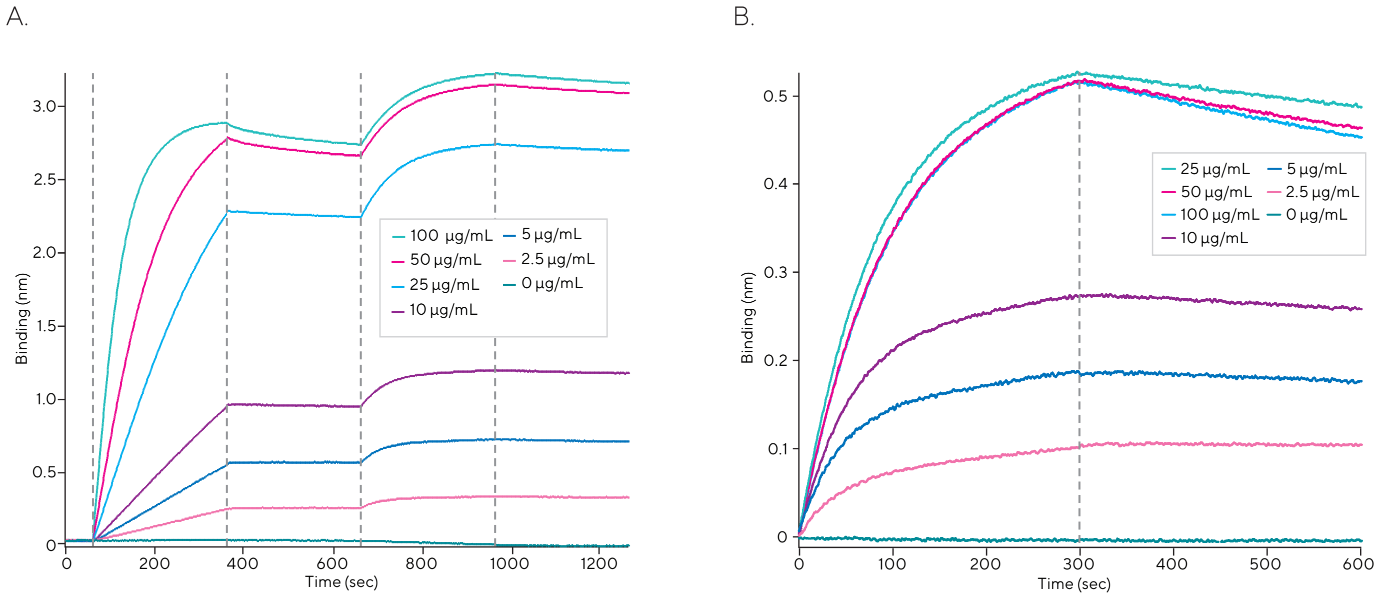

Note. A) Raw sensorgram data for ligand loading optimization experiment on the Octet® BLI system. Biotinylated anti PSA antibody (Fitzgerald) was loaded onto Streptavidin Biosensors at several concentrations in 1X Kinetics Buffer (Sartorius). After a baseline step, PSA antigen (Fitzgerald) was associated to the immobilized ligand at a concentration of 200 nM, followed by dissociation step in 1X Kinetics Buffer. B) Aligned sensorgram traces showing association and dissociation steps. The difference in analyte signal at each ligand concentration can be easily observed when data is aligned.

Figure 10: *Sensorgram Data Illustrating Varying Ranges of Analyte Concentrations.*

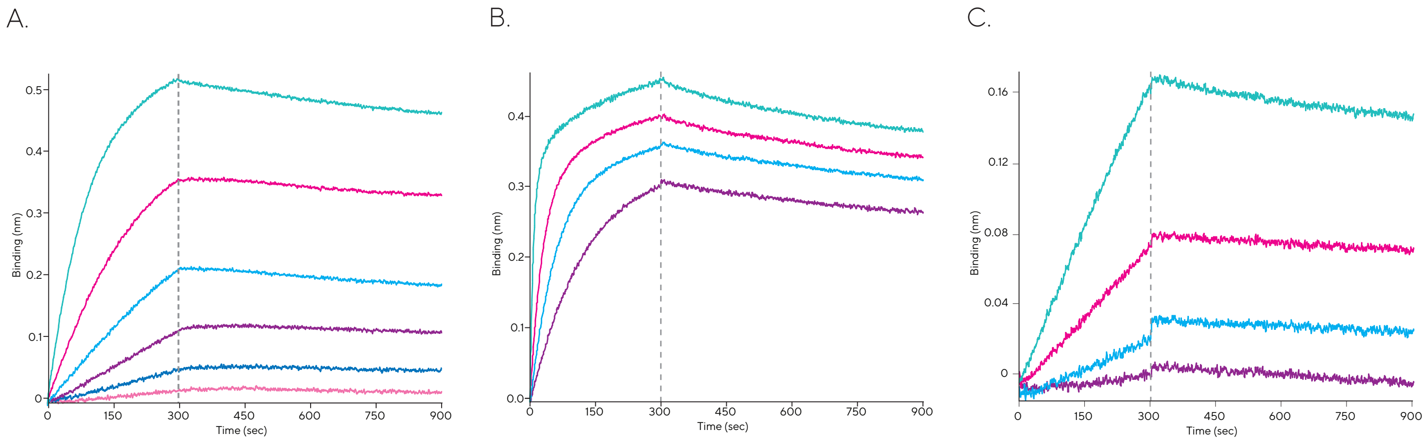

Note. A) Ideal range of analyte concentrations for a kinetics experiment, with signals spanning the dynamic range of the assay. Curve fits generated using such data will likely give reliable kinetic constants. B) The range of analyte concentrations utilized is too high. Note that the curves are close together and exhibit heterogeneity most likely due to non-specific binding. The lower range of concentrations is not represented, which tend to give the most accurate data. C) The range of analyte concentrations here is too low, and data is limited by mass transport.

that used in the ligand immobilization step. Switching to a different buffer solution can create matrix effects due to non-specific binding of new buffer components, significant changes in refractive index or drift from dissociation of ligand molecules from the surface (especially for capture biosensors) that must be allowed to equilibrate. It is important to establish a stable baseline with minimal signal drift before proceeding to the association step. An unstable baseline will impact measurement of the dissociation phase, especially if the baseline drift is high relative to the dissociation rate or is drifting in a non-linear fashion. Baseline drift also impacts maximum binding capacity, or R_{max}. R_{max} is a factor in calculating rate constants and K_D in both kinetics and equilibrium analysis, so it is important that R_{max} remain constant throughout the association and dissociation phases.

Association Step

In the association step, the binding interaction of the analyte to immobilized ligand is measured. For screening purposes or qualitative analyses, measuring binding curves for a single analyte concentration is often sufficient. However, when reliable, accurate kinetic constants are required, a dilution series of at least four analyte concentrations should be measured in the association step. The analyte dilution series measured should ideally range from a concentration of about 10–20*K_D down to about 0.1*K_D, using 2-fold or 3-fold dilutions. Since K_D is defined as the concentration of analyte at equilibrium that occupies 50% of the available binding sites on the biosensor surface, this range will assure that the assay will span from about 90% R_{max} down to the limit of detection. Running several concentrations will also show how well the fitted binding model applies over a concentration range around the K_D. If the K_D of the interaction is not known, either through experimentation or literature, an initial analyte scouting step is recommended. In this case, run the assay using a few analyte concentrations that span a wide range in order to approximate the K_D prior to running a full characterization.

Figure 10A shows kinetic data from an experiment using an optimal range of analyte concentrations. The binding signals span the dynamic range of the assay from near the limit of detection to just below saturation. The even spacing between the association curves indicates the ligand on the biosensor has not been saturated with analyte. If the analyte concentrations tested in a kinetic assay are too high, ligand binding sites can be saturated, increasing the potential for artifacts and non-ideal behavior related to non-specific binding or aggregation (Figure 10B). The lower range of analyte concentrations is not represented, though this is the range where the most accurate data are produced. If analyte concentrations are too low, however, signal may be weak and/or the resulting binding rates may be diffusion limited (Figure 10C).

In practice, binding signals may sometimes be too low at analyte concentrations near or below the K_D value. For example the observed binding rate will be very low for a high-affinity binder run at low concentrations. This low rate will affect the amount of binding that can be observed in a five or ten minute association step. If binding signal below the K_D is not measurable, it is best to run a dilution series beginning at 100X K_D and titrate down using two- or three-fold dilutions until there is no measurable signal. This

Table 4: *Effect of Dissociation of Molecules from the Biosensor on Concentration in Buffer Solution.*

Extent of Dissociation	# Molecules Going into Solution	Moles Going into Solution	Volume/Well (µL)	Molarity in Solution (M)	pM at End of Dissociation
100% dissociation (unlikely unless very weak interaction)	1.00E+09	1.66E-15	200	8.30E-12	8.3
10% dissociation	1.00E+08	1.66E-16	200	8.30E-13	0.83
1% dissociation	1.00E+07	1.66E-17	200	8.30E-14	0.083

Note. The number of molecules coming off the biosensor is negligible even when the same well is used for multiple samples in the dissociation step.

strategy will enable testing over a range of concentrations and kinetic and binding constants measured this way will still be valid.

The association step should be run long enough to see some curvature in the data traces, but not so long that the curves flatten out for an extended period of time. In general, a five to ten minute association step is recommended. For fast-binding molecules, typically a 1–2 minute association time is adequate. However, for slower reactions, a longer association step may be necessary. Avoid running the association step for too long and allowing binding curves to reach equilibrium, as this leaves opportunity for weaker, non-specific interactions to occur.

When running a full kinetic profile with several analyte concentrations, data are analyzed globally by fitting both association and dissociation phases for several analyte concentrations simultaneously using the same set of rate constants. Global analysis of a wide range of analyte concentrations provides robust analysis and accurate estimation of binding constants. Knowing the correct analyte concentration is critical, as this value also has a direct impact on calculated constants.

Dissociation Step

In the dissociation step, the biosensor is dipped into buffer solution that does not contain analyte. With no analyte present, the free concentration of analyte in solution drops to zero and bound complex on the surface of the biosensor dissociates. It is recommended that the dissociation step be run in the same microplate well as the baseline for each sample. Running these two steps in the same buffer in the same well can prevent step artifacts that may result from subtle differences in buffer, volume or shape of the well. While these artifacts can be corrected for, it is best to minimize them for accurate data analysis.

The dissociation step can be run in the same well for every sample in an assay, as long as the buffer matrix is identical for each sample. Therefore only one column of a microplate needs to be reserved for dissociation buffer per plate of analyte samples. This leaves room to analyze more samples in parallel, increasing throughput and efficiency. It is acceptable to dip into the same well of buffer for every dissociation step because the number of molecules coming off the biosensor surface is negligible, even when the biosensor is saturated and dissociation rate is high, as shown in Table 4. It may be beneficial to use separate wells for dissociation of each sample in cases where off-rates are extremely fast and molecules dissociate fully from the biosensor.

The dissociation step should be run long enough to observe decay in the binding response, meaning the length of the dissociation step will depend on the affinity of the interaction. A high affinity interaction can be difficult to analyze if the dissociation curve is measured for too short a time. Ideally, at least 5% of complex should dissociate for robust analysis. (Refer to Table 3 for the relationship between affinity and complex half-life). An important advantage of using an Octet® BLI system for kinetic measurements is that long dissociation steps can be performed for multiple analyte concentrations in parallel rather than one sample at a time, greatly minimizing experimental run time.

Non-specific Binding

Biological molecules tend to interact with surfaces; therefore non-specific binding is a concern with any assay under any conditions. Non-specific binding can occur as a result of many factors, such as cell culture media components, BSA or serum proteins, or charged species in the sample or buffer. The biocompatible layer on the biosensors greatly mitigates non-specific binding, however some consideration must be taken to minimize buffer effects.

Pre-hydration of biosensors is required before running an assay. If pre-hydration is performed for at least 10 minutes in media or buffer that matches that of the corresponding samples, non-specific binding can be greatly reduced. Be sure to use the same buffer for baseline, association and dissociation steps during the assay.

When non-specific binding occurs, proper assay optimization and buffer selection can minimize the effects. Modifying the assay buffer can reduce such binding. In most assays measuring protein-protein interactions, blocking agents such as BSA (up to 1–2%) and/or non-ionic detergents such as Tween-20 (up to 0.09%) can be added. Other blocking agents such as casein, PEG or gelatin can also be substituted. Salt concentration can also be modified. Raising the salt concentration to 150–500 mM (physiological concentrations and higher) can increase assay stringency and therefore reduce non-specific binding.

Always include a reference sample with every kinetics experiment to allow subtraction of background signal and assay drift. A reference sample is run in the association step using a ligand-loaded biosensor, and should consist of assay buffer minus the target analyte. Double referencing with both a reference sample and a reference biosensor can be performed when background signal is an issue, or in small molecule binding assays where the signal is very small in relation to noise. Reference biosensors are typically loaded with a non-active protein that is similar to the ligand, and run through the assay with the same analyte samples as the ligand-loaded biosensors. The reference biosensor is used to subtract non-specific binding of analyte to the biosensor.

Biosensor Regeneration

Although Dip and Read biosensors are disposable and cost effective for single-use applications, many are also regenerable. In some cases, especially in kinetic screening, it may be advantageous to assay several samples using the same ligand-coated biosensor. This practice can save some cost on biosensors; however, these savings should be carefully weighed against the costs involved in optimizing regeneration conditions.

For kinetic applications, Streptavidin and AR2G Biosensors can be regenerated down to the level of the immobilized ligand by removing bound analyte under disrupting conditions. Efficient regeneration requires removing the bound analyte without affecting ligand activity. However, the conditions that effectively disrupt ligand-analyte binding are protein dependent. Similarly, immobilized ligands vary significantly in their ability to retain activity after regeneration. The appropriate regeneration protocol for a particular ligand-analyte protein pair must be determined experimentally. Most antibody-protein interactions can be disrupted by a series of short incubations in low pH buffer (pH 1–4), such as 10 mM glycine, (pH 1.5–2.0), followed by neutralization in assay buffer. However, if the biosensor surface is not efficiently regenerated with low pH buffer, other conditions may be tested such as high salt concentrations, detergent, or high pH buffer. There are different modes of interaction at work between different ligand-analyte pairs, such as hydrophobic forces, van der Waals forces, and ionic binding. An understanding of the nature of the proteins involved and the type of non-covalent forces that dominate the interaction is important in developing an effective regeneration protocol.

For successful regeneration, the following conditions must be met:

- Biosensor surface chemistry must be stable under the regeneration conditions.
- The immobilized ligand must be stable under the regeneration conditions and retain activity over multiple regeneration cycles.
- Ligand-analyte protein interaction must dissociate fully during regeneration.

The number of regeneration cycles that a ligand can withstand and the efficiency of the regeneration also greatly depend on the proteins being disrupted. Some ligands can be regenerated ten or more cycles, while others tolerate far fewer cycles or cannot be regenerated at all. A table of suggested biosensor-compatible regeneration buffers is shown in Table 5. The Octet® BLI platform's high-throughput format provides a flexible platform for assays that incorporate regeneration as well as efficient screening of regeneration conditions.

There are many possible ways to configure a screen for regeneration conditions. The ligand can be immobilized onto eight biosensors to screen up to eight regeneration solutions in parallel to take advantage of the Octet® BLI platform's throughput capacity. Run the assay with an analyte concentration of at least 10X*K_D, using up to ten regeneration cycles (Figure 11). In general, several short exposures (*e.g.* 3 to 5 exposures, 5 seconds each) to the regeneration buffer in a cycle are more successful than a single, longer exposure.

Table 5: *Table of Suggested Biosensor-compatible Regeneration Buffers.*

Biosensor	Regeneration Reagent	Maximum Recommended Exposure Time
Streptavidin	HCl pH 0.5–1.5	15 minutes
Streptavidin	NaOH pH 10–11	15 minutes
Streptavidin	10 mM Glycine (pH 1–3)	15 minutes
Streptavidin, AR2G	NaCl, 1–5 M	15 minutes
Streptavidin	MgCl2, 0.1–1 M	15 minutes
Streptavidin	Tween 20, 0.1–0.5%	15 minutes
Streptavidin	SDS, 0.005–0.01%	15 minutes
Streptavidin	Phosphoric acid, 50–500 mM	15 minutes
Streptavidin	EDTA, 25–100 mM	15 minutes
Streptavidin	Triton X-100, 0.1–0.5%	15 minutes
AR2G	Acetate buffer, 10–100 mM (pH 0.5–3)	15 minutes
AR2G	Citrate buffer, pH 2	15 minutes
AR2G	KOH, pH 9–11	15 minutes
AR2G	Phosphoric acid, pH 2	15 minutes
AR2G	SDS, up to 0.5%	15 minutes
AR2G	$MgCl_2$, up to 4 M	15 minutes
AR2G	HCl, 1 mM	15 minutes
AR2G	Ethylene glycol 25% or 50%	15 minutes
AR2G	0.15 M oxalic acid, 0.15 M H_3PO_4, 0.15 M formic acid and 0.15 M malonic acid *	15 minutes
AR2G	0.2 M ethanolamine, 0.2 M Na_3PO_4, 0.2 M piperazine and 0.2 M glycine **	5 minutes
AR2G	0.46 M KSCN, 1.83 M $MgCl_2$, 0.92 M urea and 1.83 M guanidine-HCl	5 minutes
AR2G	Mixture of CHAPS, zwittergent 3-12, Tween 80, Tween 20 and Triton X-100, each at 0.3 %, 20 mM EDTA	5 minutes

Note. References: *RC Chatelier, TR Gengenbach, HJ Griesser, BM Brigham, and DJ O'Shannessy. A general method to recondition and reuse BIAcore sensor chips fouled with covalently immobilized protein/peptide. *Anal. Biochem.*; (229): 112–118; 1995. **K Andersson, D Areskoug, and E Hardenborg. Exploring buffer space for molecular interactions. *J.Mol.Recognit.*; (12): 310–315; 1999.

If the regeneration is successful, the analyte binding curves from each cycle will overlay with minimal change in binding capacity when compared to earlier binding cycles (Figure 12B).

Figure 12A shows the overlay of signal response for association of the analyte after each regeneration step. The signal decreases dramatically with each regeneration cycle. Figure 12B shows the overlay of analyte association for ideal regeneration conditions, where the signal does not decrease with the number of regeneration cycles. In some cases, binding capacity may decrease during the first regeneration cycle but stabilize through subsequent cycles. This decrease may be due to many factors including loss of a small amount of immobilized protein during the first exposure to regeneration buffer. To minimize the impact of this initial change on final data, it is common practice to run a pre-conditioning step before the assay, where one cycle of regeneration is performed.

Figure 11: *Real-Time Results from Regeneration Scouting and Validation Experiment Run on the Octet® R8 BLI System.*

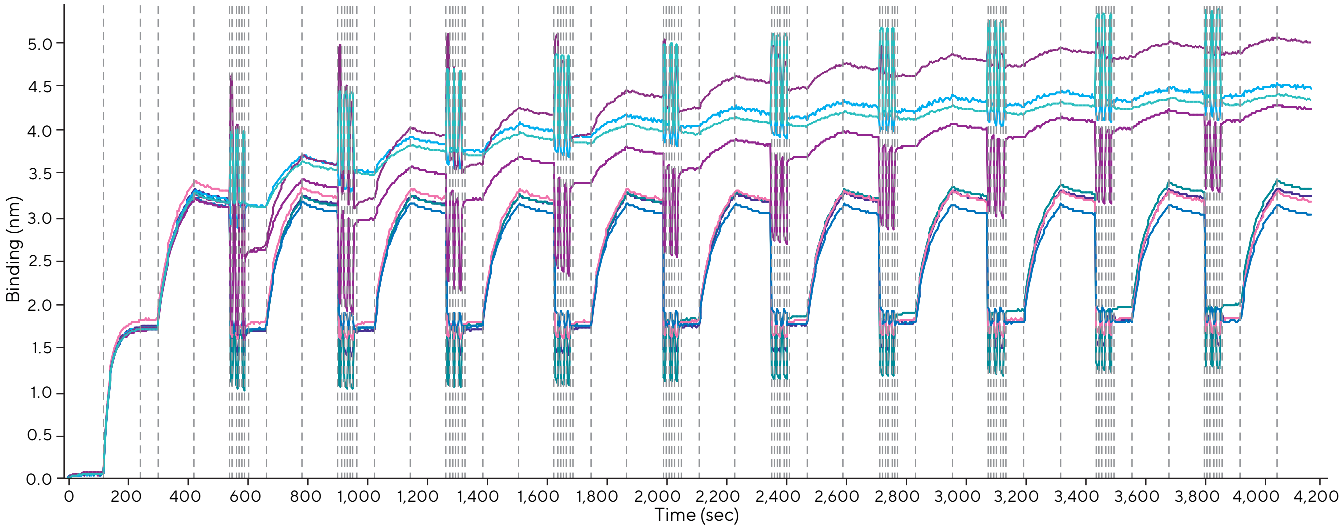

Note. Data shown are from eight Streptavidin Biosensors (A1–H1) each corresponding to a different regeneration solution. Biotin-Protein A was immobilized, followed by 11 cycles of binding to human IgG with regeneration.

Figure 12: *Examples of Analysed Regeneration Cycles from Data in Figure 11.*

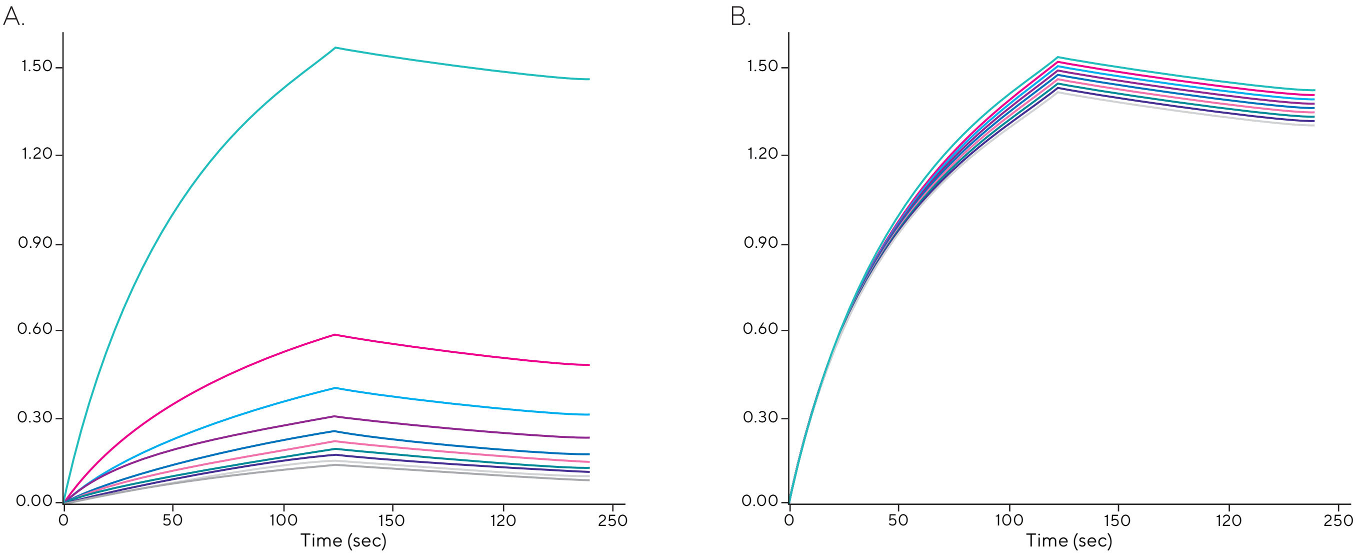

Note. Aligned sensorgram traces showing association and dissociation steps for two of the eight regeneration conditions tested in the experiment in Figure 11. Chart A shows data from non-optimal regeneration reagent (NaOH, pH 11) where association signal declines with each subsequent regeneration cycle. Chart B shows optimal regeneration condition (10 mM glycine, pH2) where binding activity of the ligand is maintained through all regeneration cycles.

For capture-based biosensors, such as Anti-human IgG Fc Capture or Anti-mouse Fc Capture, the surface can be regenerated to the level of the original surface chemistry. Both the captured ligand and the analyte are removed during regeneration, which is typically performed using 10 mM glycine (pH 1.7) regeneration buffer. Three to five cycles are performed consisting of a 5-second incubation in regeneration buffer followed by 5-second incubation in assay buffer. The biosensor can then be re-loaded with ligand for a new kinetic assay. Depending on the protein being captured, however, regeneration buffer formulation may require optimization. For recommended regeneration conditions for a specific capture-based biosensor, refer to the corresponding technical note on the Sartorius web site.

Running a Binding Kinetics Assay

Steps to set up a kinetics assay are simple and straightforward using the Octet® BLI Discovery Software and microplate format. Octet® BLI systems offer great flexibility in kinetic assay design and many options are available depending on the format and the instrument being used. Detailed instructions on setting up an assay using the Octet® BLI Discovery Software can be found in the Octet® BLI Discovery Software User Guide.

A typical assay protocol runs as follows:

1. Prepare buffers, regeneration solution, ligand and analyte samples.
2. Hydrate the biosensors for at least 10 minutes. Hydration buffer should match the buffer used throughout the assay as closely as possible.
3. Prepare the assay plate, filling columns of wells with buffers, regeneration solution, ligand and interacting protein samples.
4. Set up the assay in the Octet® BLI Discovery Software: define plate layout, define assay steps and assign biosensors.
5. Equilibrate both the hydrated biosensor assembly and the assay plate for 10 minutes on the Octet® BLI system. Equilibration allows the biosensors to fully hydrate and the assay plate to reach a stable temperature.
6. Run the assay.
7. Perform data processing and analysis.

A typical sample plate layout and assay summary is shown in Figure 13.

A column format is recommended for a typical large molecule kinetics assay, where sample dilutions are made down the columns of the microplate. Samples are measured simultaneously in each column so that sample dilutions are measured in parallel. Biosensors are replaced or regenerated between different analytes being tested. In a row format, sample dilutions are made across the microplate row. This format typically is used in small molecule kinetic assays when affinities are low and dissociation usually is rapid and complete, where dissociation is usually rapid and complete, so that regeneration of biosensors between sample dilutions is not required. Figure 13B shows a typical kinetic assay setup with recommended step times. Note that the same column of buffer is used for the baseline and dissociation steps.

Baseline, association and dissociation steps must be run in sequence. Shaking speed and assay step lengths can be changed depending upon the strength and speed of the interaction. Increasing the shake speed will increase the sensitivity of the assay, and is recommended for weaker

Figure 13: *A) Plate Layout Diagram for Standard Kinetics Assay. B) Summary of Standard Kinetics Assay wth Suggested Step Times.*

A.

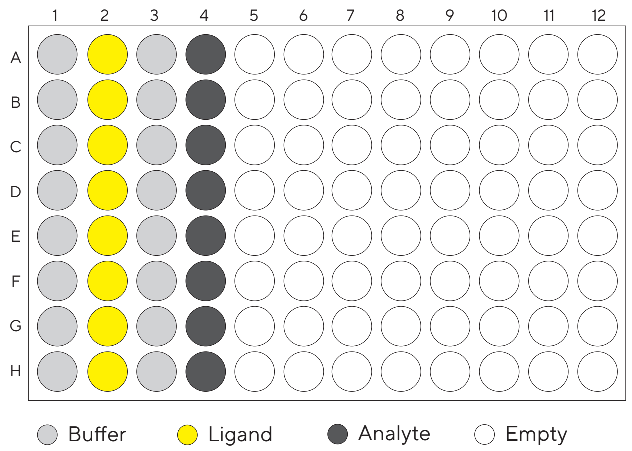

B.

Step #	Step Name	Time (sec)	Flow (RPM)	Sample Plate Column
1	Equilibration	60	1000	1
2	Loading	300–600	1000	2
3	Baseline	180–600	1000	3
4	Association	300–600	1000	4
5	Dissociation	300–3600	1000	3

binders or lower concentrations of reagent. If an interaction is very rapid, or if a reagent is used at high concentrations, a slower shake speed of 400 RPM is recommended. The association step time can be increased for a slow interaction or a weak binder to enhance binding signal. The dissociation step should be longer for high affinity binders. In general, step lengths of 1 minute for baseline, 5 minutes for association and 10 minutes for dissociation are a good starting point, however some optimization may be required. For accurate calculation of binding and affinity constants, at least 5% of the complex must dissociate. A high affinity binding pair, for example K_D < 1 nM, may require a longer dissociation time of 30 minutes or more. Overall run time should be no longer than 3 hours, as sample evaporation from the microplate wells may begin to impact results. Octet® R8 platform however, is capable of utilizing a microplate evaporation cover that enables unattended run times up to 12 hours.

Some important considerations to remember when running a kinetic assay:

- The buffer used for biosensor pre-hydration, baseline and dissociation steps must match the matrix of the analyte sample as closely as possible.
- Select a ligand loading concentration that does not saturate the biosensor, but still provides a strong analyte signal. Ideally, for a 150 kDa antibody, the signal in the loading step should reach about 1.0 nm after 10 minutes of loading.
- Run a 2-fold or 3-fold dilution series of at least four concentrations of the analyte, starting at 10-fold above the K_D.
- The same buffer wells should be used for the baseline and dissociation steps. This will minimize artifacts and enable use of the inter-step correction feature in the Octet® Analysis Studio Software.
- Run a reference sample, or negative control, consisting of buffer-only without analyte.
- Be sure the baseline is stable (has a flat signal with minimal downward drift) before proceeding to the association step.

Running an assay at 30°C is recommended for optimal results, since binding to the biosensor is sensitive to fluctuations in temperature. By working at a few degrees above ambient, a consistent temperature can be maintained through the course of the assay.

Analysis of Binding Kinetics Data

Binding kinetic sensorgrams contain a great deal of information describing an interaction between molecules. A sensorgram can answer simple questions about the presence or absence of a binding interaction between molecules, and enable complex analyses that fully characterize an interaction and extract binding rate and equilibrium binding constants. The Octet® Analysis Studio Software is a powerful, yet simple-to-use program for analyzing kinetic data. In this section, we will describe features of the data analysis software, steps in analyzing data, and considerations for interpreting results.

Kinetic data are interpreted based on a mathematical model of the interaction, from which kinetic and equilibrium binding constants can be calculated based on rates of association and dissociation. This type of analysis can be performed either locally, where kinetic parameters are determined based on a single analyte concentration, or globally, where constants are derived simultaneously from all analyte concentrations available. Alternatively, the equilibrium dissociation constant (K_D) can be determined using data at equilibrium from each available analyte concentration using steady state analysis.

General steps for performing analysis of kinetic data are as follows:

1. Load acquired data into the Octet® Analysis Studio Software. Process the data in the Processing window by specifying methods for reference subtraction, y-axis alignment, inter-step correction and Savitzky-Golay filtering.
2. Analyze the data in the Analysis window by specifying steps to analyze, selecting curve fit model, fitting method (local or global) and window of interest.
3. Export the analyzed data by clicking "Save Report" to generate a detailed report in Microsoft® Excel®.

Recognizing Non-ideal Binding Behavior

It is important to examine the real-time sensorgram curves prior to data analysis, as they reveal information about the nature of the interaction as well as the quality of the data. Ideally, only the interaction under study will contribute to the binding profile, however, additional factors can also

affect the shape of a binding curve on a sensorgram. When complex, non-optimal binding occurs due to multiple types of interactions taking place on the biosensor, the resulting sensorgram will show characteristics that deviate from the classical 1:1 bimolecular interaction. Figure 14 shows examples of ideal (homogeneous) and non-ideal (heterogeneous) binding profiles. The association phase of the blue heterogeneous curve is clearly biphasic, rising steeply at first and then continuing to rise at a slower pace where a 1:1 interaction would be expected to level off at equilibrium in a smooth monophasic binding curve. The biphasic nature of the blue curve is also reflected in the dissociation step, where a fast initial decay is followed by slower decay later in the step. In some cases of non-ideal binding (not illustrated in the figure), the dissociation curve signal stabilizes above the pre-association baseline signal. These binding curve characteristics indicate a complex binding event, which can affect reliable curve fitting and data analysis.

While an interaction displaying non-ideal behavior may be easily fit using a more complex binding model such as 2:1 or 1:2 binding, it is best not to choose a more complex curve fitting model without significant justification. Unless the interaction is known to be complex, the best approach is to optimize assay conditions until the profile displays 1:1 binding characteristics.

A complex, non-ideal binding profile in a kinetic assay can be caused by a number of factors. Proper assay optimization can often alleviate the apparent heterogeneity and bring the interaction back to its expected binding behavior. One major component in complex binding behavior may be non-specific binding to the biosensor. Techniques for mitigating this type of binding are discussed in the Developing a Kinetics Assay section. When analyte concentrations far above the K_D are used, and/or ligand density on the surface is high, weaker non-specific interactions tend to come into play. Lowering the analyte concentration range in the assay or eliminating higher concentration data traces from the analysis can bring the interaction closer to an ideal curve fit. Lowering the ligand density on the surface, either by reducing ligand concentration during loading or by decreasing the loading step time, can have a similar effect.

Strategies for Correcting Complex Binding Behavior

- Use capture-based approach.
- Lower ligand density.
- Lower analyte concentration.
- Check reagent stability and quality.
- Modify assay buffer by adding blocking agents, detergent.
- Shorten association step time.

Figure 14: *Complex, Non-Optimal Binding Will Result in Sensorgram with Characteristics that Deviate from Classical 1:1 Interaction.*

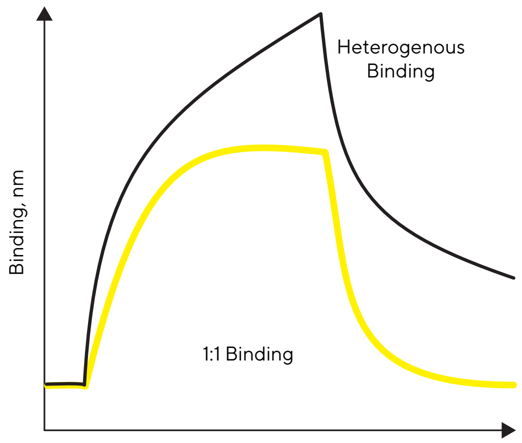

Note. Heterogeneous binding is characterized by biphasic curves that fail to equilibrate in the association step and often do not return to baseline signal in the dissociation step.

Structural heterogeneity in the ligand or analyte, occurring either in the original samples or as a result of artifacts in the assay, may contribute to a complex binding profile. Poor quality ligand or analyte samples can create heterogeneous binding effects. The presence of partially active or inactive proteins and aggregates will impact a kinetic profile, causing non-optimal binding. Use of fresh, high quality reagents that have been stored properly and tested for stability is always recommended. The chosen method of immobilization can cause physical effects that may lead to ligand heterogeneity. For example, when performing covalent immobilization on an Amine Reactive Biosensor, amine groups near the binding site may result in sub-populations of ligand with different binding characteristics. This effect can be prevented by using a capture-based immobilization strategy where the ligand is oriented on the surface (see Developing a Kinetics Assay section).

When ligand density on the surface is very high and/or the interaction has a fast on-rate, rebinding of analyte can potentially occur during the dissociation phase. This is a commonly observed effect with all biosensor techniques that involve ligand bound to a solid support. Rebinding occurs when an analyte molecule dissociates from one ligand molecule only to immediately bind to a neighboring molecule on the surface. The effect is sometimes called analyte "walking" and can lead to complex binding kinetics and under-estimation of off-rates. The best solution is to lower the ligand density on the biosensor either by reducing the concentration of ligand during the loading step, or by shortening the loading step time.

When a binding pair exhibits complex binding behavior even when a 1:1 interaction is expected, proper choice of assay format and careful assay development can often eliminate artifacts and bring the apparent binding interaction to be consistent with a 1:1 kinetic profile.

Processing Parameters

After a data set has been acquired and loaded into the data analysis software, it must be processed in the Processing window. This window provides tools for correcting binding curves using various reference subtraction and alignment options. The data processing steps specify how to reference the data and produce the final binding curves.

- Reference subtraction is performed in this stage of the analysis. Reference samples and/or biosensors can be selected in the Sensor Selection window and method of subtraction chosen in the Raw Data view.
- To fit curves correctly, they must be aligned to a common reference point on the Y axis:
  - Alignment along the X axis is achieved during the assay due to the parallel movement of all biosensors.
  - Alignment along the Y axis is achieved using Align Y Axis feature by specifying both a step and time with which to execute the alignment.
  - The time range from the specified step will be used to calculate an average and that average will then be set to y=0.

For example, for alignment to the baseline, select Baseline and specify the time within the baseline step to set to an average y=0. Typically, the Y-axis is aligned to the last five seconds of the baseline step, where the signal has stabilized.

- The Interstep Correction feature corrects any misalignment between two steps resulting from assay artifacts.
  - Align to Dissociation—Moves the association step on the Y axis to align the end of the association step with the beginning of the adjacent dissociation step.
  - Align to Baseline—Moves the association step on the Y axis to align the beginning of the association step with the end of the adjacent baseline step.

For the most effective interstep correction, the baseline and dissociation steps must be run in the same microplate well during the assay. Avoid using interstep correction for binding pairs with very fast on- and off-rates, as some kinetic information may be lost.

- Savitzky-Golay filtering removes high-frequency noise from the data. Its use is optional, but is recommended for data collected using standard acquisition rates.

Analysis Window: Choosing a Curve Fit Model

In the Analysis window, processed data can be analyzed by fitting to one of the available curve fit models. Fitting the experimental data to a model involves some consideration. Many interactions studied do not fit a simple 1:1 binding model. When deviation from this model is a function of the type of interaction, rather than an experimental artifact, additional pre-programmed curve fitting models are available in the Octet® Analysis Studio Software:

- 2:1 HL Model (heterogeneous ligand).
- 1:2 Bivalent Analyte Model.
- Mass Transport.

While the 1:1 binding model assumes the interaction between ligand and analyte follows pseudo-first-order kinetics, the alternative models can be used to calculate binding constants for more complex interactions. The appropriate kinetic binding model for an interaction should be chosen based on knowledge of the interaction and the molecules involved, valency and predicted stoichiometry of binding or estimated binding constants. It can be tempting to choose a model based based upon best empirical fit to the data. More complicated models feature more variables and degrees of freedom, and tend to offer statistically better fits. However, the 1:1 model should be used unless the interaction is known to be more complex, or the goal is to attempt to understand a mechanism of interaction. If the fit is poor, it is possible that components not originating from the interaction of interest are impacting the data. Assay artifacts caused by non-specific binding, overloading of the biosensor, poor reagent quality, inappropriately timed assay steps, or unstable baselines can affect binding curves. Such variables must be eliminated by further optimizing the experimental design before accurate curve fitting can be accomplished.

1:1 Binding Model

In a 1:1 bimolecular interaction, both the association and dissociation phases display a time-resolved signal that is described by a single exponential function. Analyte molecules bind at the same rate to every ligand binding site. The association curve follows a characteristic hyperbolic binding profile, with exponential increase in signal followed by a leveling off to plateau as the binding reaches equilibrium. The dissociation curve follows single exponential decay with signal eventually returning to baseline. The full fitting solution for a 1:1 binding is:

Association phase:

$$y = R_{max} \frac{1}{1 + \frac{k_d}{k_a * [\text{Analyte}]}} \left(1 - e^{-(k_a * [\text{Analyte}] + k_d)x}\right)$$

Dissociation phase:

$$y = y_0 e^{-k_d (x - x_0)}$$

$$y_0 = R_{max} \frac{1}{1 + \frac{k_d}{k_a * [\text{Analyte}]}} \left(1 - e^{-(k_a * [\text{Analyte}] + k_d)x_0}\right)$$

An Example Of Kinetic Data Following A 1:1 Interaction Is Shown In Figure 15.

Heterogeneous Ligand Model

The 2:1 heterogeneous ligand model assumes analyte binding at two independent ligand sites. Each ligand site binds the analyte independently and with a different rate constant. Two sets of rate constants are given, one for each interaction:

$$A + B1 \underset{k_{d1}}{\overset{k_{a1}}{\rightleftharpoons}} AB1$$

$$A + B2 \underset{k_{d2}}{\overset{k_{a2}}{\rightleftharpoons}} AB2$$

where A represents the analyte and B represents the immobilized ligand. Mathematically, the equation used to fit a 2:1 binding interaction is a combination of two 1:1 curve fits, with an additional parameter to account for percentage of binding contributed by each interaction. Figure 16 shows an example of fitted data using the 2:1 heterogeneous ligand binding model. Note the biphasic nature of the association and dissociation curves. In the association phase, the initial fast on-rate is followed by a slower on-rate, with the signal continuing to increase rather than reaching equilibrium. In the dissociation phase, an initial fast dissociation is followed by a slower off-rate, with the higher concentration curves failing to return to baseline.

Figure 15: *Analyzed Data Illustrating A 1:1 Binding Interaction.*

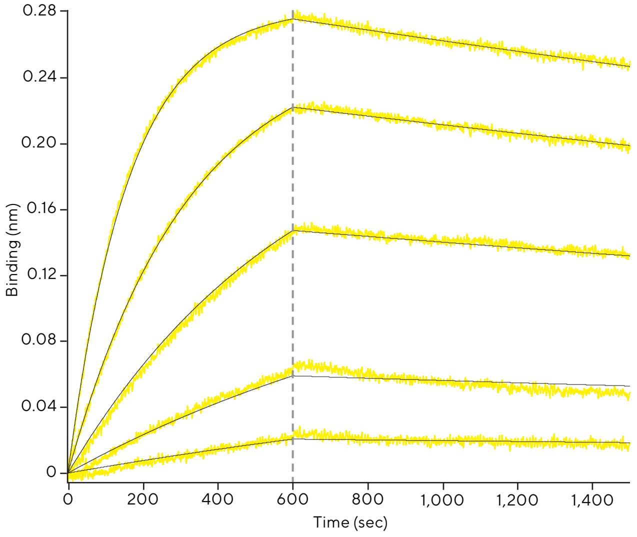

Note. Black lines represent data traces, curve fits are in yellow.

Figure 16: *Analyzed Data Fit Using the 2:1 Heterogeneous Ligand Binding Model.*

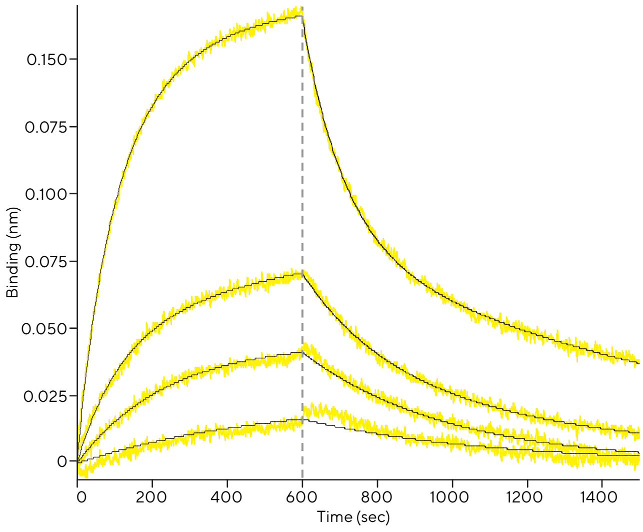

Note. Curves are biphasic, indicating that more than one interaction is occurring.

Mass Transport Model

In a fluidics-based system, samples pass over the biosensor surface via laminar flow, where frictional forces from the sides of the tubing and the surface of the biosensor slow the velocity of the liquid close to the surface. In such a system, the decreased flow rate immediately adjacent to the biosensor inhibits efficient exchange of analyte molecules from the surface to the bulk solution. At low concentrations, analyte molecules present near the biosensor surface can bind to the ligand faster than they can be replaced in the surrounding solution. When this occurs, the binding rate becomes dependent on supply of analyte molecules rather than the actual kinetics of the interaction. The shape of the binding curve will be determined by the rate at which the analyte diffuses to the surface, and will change with flow rate. As flow increases, so does the apparent binding rate, as the supply of analyte molecules available to bind increases.

$$A_{bulk} \underset{k_m}{\overset{k_m}{\rightleftharpoons}} A_{surf} + B \underset{k_d}{\overset{k_a}{\rightleftharpoons}} AB$$

Often mass transport limited curves are often less steep than expected, and can appear as straight lines (see Figure 10C).

Mass transport limited data can be fit using the Mass Transport binding model, which fits the binding of the analyte taking into account two steps: 1) rate of transport of the analyte from the bulk solution to the surface (km), and 2) interaction of the analyte with the ligand. Diffusion limitation occurs when km is much smaller than ka[B]

$$\frac{dR}{dt} = \frac{k_a * [Analyte]}{1 + \frac{k_a}{k_m}(R_{max} - R)}(R_{max} - R) - \frac{k_d}{1 + \frac{k_a}{k_m}(R_{max} - R)} R$$

Agitating the sample plate in the Dip and Read format creates a turbulent flow over the biosensor which is not subject to laminar forces and is highly efficient at replacing volume close to the surface of the biosensor. If mass transport effects are an issue, the supply of analyte to the surface must effectively be raised. This can be accomplished by reducing the level of immobilized ligand, or increasing the shaking speed during the assay to increase flow rate.

1:2 Bivalent Analyte

The 1:2 Bivalent Analyte model fits the binding of one bivalent analyte to a monomeric immobilized ligand. Kinetic parameters are calculated for two interactions (k_{a1}, k_{a2}, k_{d1}, k_{d2}, K_{D1}, K_{D2}).

$$A + B \underset{k_{d1}}{\overset{k_{a1}}{\rightleftharpoons}} AB \longrightarrow AB + B \underset{k_{d2}}{\overset{k_{a2}}{\rightleftharpoons}} AB$$

This model assumes that because of limited distance between two adjacent binding sites on the surface, the bivalent analyte can form a bridged complex (Figure 17). This interaction is linked, meaning that the formation of AB2 complex cannot occur before the formation of AB, and AB cannot dissociate before the dissociation of AB2. This avidity effect results in a slower apparent dissociation rate than would be expected if the interaction followed a 1:1 binding profile.

Figure 17: *Illustration of Bivalent Analyte Binding.*

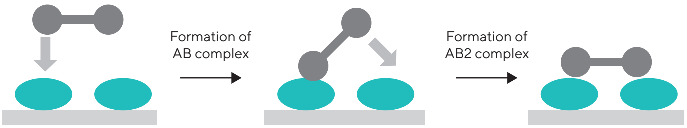

Note. Due to limited distance between adjacent ligand molecules on the surface of the biosensor, a bivalent analyte can form a bridged complex. This effect creates a lower apparent dissociation rate than would be expected. The bivalent analyte binding model accounts for avidity when calculating on- and off-rates.

Two sets of rate constants and K_D values are reported using the 1:2 bivalent analyte model. The first set of values reflects the binding due to the affinity of the interaction. The second set of values represents binding due to avidity. Three equations are used to fit bivalent analyte curves, the first describing association of A to B, the second is the association of AB to B, and the third describes dissociation of the AB2 complex.

$$\frac{dB}{dt} = -(2k_{a1} * [A] * B - k_{d1} * AB) - (k_{a2} * AB * B - 2k_{d2} * AB_2)$$

$$\frac{dAB}{dt} = (2k_{a1} * [A] * [B] - k_{d1} * [AB]) - (k_{a2} * [AB] * [B] - 2k_{d2} * [AB_2])$$

$$\frac{dAB_2}{dt} = (k_{a2} * AB * B - 2k_{d2} * AB_2)$$

Changes in the assay format and conditions can reduce avidity effects in a binding interaction. One approach is to lower the density of the immobilized ligand by decreasing ligand concentration or decreasing the loading step time. A lower ligand density will effectively increase the distance between molecules on the surface, minimizing the likelihood of a bound analyte reaching adjacent molecules. This approach may require some optimization because lowering the ligand density will decrease the sensitivity of the assay. Alternatively, reversing the assay orientation so that the bivalent molecule is immobilized on the surface will eliminate the possibility of analyte bridging. The 1:2 binding model is useful when the bivalent molecule cannot be captured due to issues related to instability under conditions of immobilization, lack of sensitivity in the opposite assay orientation, reagent availability, or if an interaction needs to be tested in multiple formats.

Local Fitting: Full vs. Partial Fit

The Octet® Analysis Studio Software provides full or partial fit options under Local Fitting. When local fitting is performed, kinetic parameters are derived individually for each analyte concentration. The full fit option assumes that an interaction is fully reversible, so that as the dissociation step time approaches infinity, all of the analyte bound to the ligand will dissociate. Since the dissociation curve will eventually reach the pre-association baseline, the rate of dissociation is extrapolated until it reaches zero signal on the Y-axis. The full dissociation option is recommended for data with a very low dissociation rate.

The partial dissociation model does not assume the signal will reach pre-association baseline. Only a portion of the analyte bound will dissociate even as the step time approaches infinity, and the rate of dissociation is fit to the measured data only. Partial dissociation can be used to fit portions of curves in data sets with significant or biphasic dissociation, however partial fitting may tend to give higher kd values. The curve fitting assumptions do not include dissociation signal decaying to zero.

Global Fit

The most accurate kinetic and affinity constants are obtained when performing a global fit using several analyte concentrations. Global fit analysis includes all binding curve data in the group using a full fit option. Fitting several curves to one set of results yields more robust and reliable curve fits. The kinetic constants that are calculated depend upon the binding model selected. R_{max} should remain unlinked by biosensor when separate biosensors are used for each individual analyte concentration. When R_{max} is linked, the theoretical maximum response is calculated assuming equal binding capacity between biosensors. Different biosensors will have slight variability in surface capacity. R_{max} can be linked if the same biosensor is used for every analyte concentration in the series. This strategy is typically used in small molecule analyses, where dissociation is rapid and complete and enables re-use of the same biosensor for a new sample concentration. In standard large molecule kinetics assays, where each sample is run on a new or regenerated biosensor, R_{max} should be unlinked to enable calculation of separate R_{max} for each sample.

Steady State Analysis

Steady state, or equilibrium analysis, may be performed when full kinetic analysis is not possible or required. The Steady State option is useful for analyzing interactions that are of low affinity or with very fast on-rates (see Table 1 for instrument dynamic ranges). If the initial slope of the binding curve is steep, it can be difficult for the software to perform accurate curve fitting. Steady State analysis is often used with protein-small molecule interactions, where on and off rates tend to be very fast and the signal fairly low. For accurate Steady State analysis results, the association binding curves must reach equilibrium for every analyte concentration in a titration. Extended association step times of 30–60 minutes may be required to reach equilibrium binding. This approach is feasible only for well-behaved binding pairs exhibiting 1:1 binding kinetics. In Octet® Analysis Studio Software, when the 'Response' option is selected, binding rate and affinity constants will be calculated based on the average signal response (nm) during the assay time specified. If the 'R equilibrium' option is selected, rate and affinity constants will be calculated based on the theoretical R_{eq} value based on the curve fits. If all curves have reached equilibrium, these two sets of values should match.

Evaluating Quality of the Fit

Once curve fitting has been performed, it is necessary to evaluate the quality of the fit and reliability of calculated binding and affinity constants. In order to assess how well the fitted curves adhere to the experimental data, refer to the general guidelines listed below.

- Visually inspect the data and determine if the fit lines conform well to the data traces. If the fit lines are far from the actual data, this indicates the fit is not ideal.
- Look at the highest and lowest concentrations of your analyte. Is the behavior of one or both of these curves different from the other concentrations? If the highest concentration(s) show greater deviation in fitting, it (they) may be excluded from the analysis. Likewise, if the lowest concentration shows little or weak response, these data may be excluded as well. Exclusion of concentrations outside of the working range of the analyte titration will improve fitting in a global analysis.
- Confirm that the apparent kinetic constants are consistent with expectations based on literature or previous experimental knowledge of the interaction.
- Residuals are calculated in the results table and plotted below the data trace in the Analysis window, and can be examined to validate the fit. Residual values should not be greater than ±10% of the maximum response of the fitted curve. The shape of the residual plot corresponds to differences between the fit curve and the experimental data and should show signals that are equally distributed above and below the mean. Higher values indicate inaccurate fitting.
- Error values are provided in the analysis table for k_a and k_d. These errors are considered acceptable if they are within one order of magnitude of the rate constant values.
- The R^2 value indicates how well the fit and the experimental data correlate. In general, R^2 values above 0.95 are considered a good fit.
- Examine Full χ^2 values. χ^2 is the sum of the squared deviations, which generally should be below 3. χ^2 is a measure of error between the experimental data and the fitted line. A smaller χ^2 indicates a better fit.

Conclusion

Here we have described the key elements to performing large molecule kinetic assays on the Octet® BLI platform and discussed strategies for producing quality kinetic data. With a wide variety of biosensors to choose from, Octet® BLI systems offer a great deal of flexibility in assay design. Proper assay development is critical to a successful assay and in assuring that an interaction can be fit to a 1:1 binding model. Considerations such as method of immobilization, assay orientation, and choice of buffer can impact results. Optimization of ligand loading density is a critical step that should be performed for every new binding pair, as an excess of ligand can lead to binding artifacts in the data. Be sure to choose an appropriate range of analyte concentrations and take steps to minimize non-specific binding heterogeneity in the data. By using proper assay development and experimental design, kinetic analysis on the Octet® BLI system will consistently yield accurate, reproducible data and reliable kinetic and affinity constants.

Germany
Sartorius Lab Instruments GmbH & Co. KG
Otto-Brenner-Strasse 20
37079 Goettingen
Phone +49 551 308 0

USA
Sartorius Corporation
565 Johnson Avenue
Bohemia, NY 11716
Phone +1 888 OCTET 75
Or +1 650 322 1360

For further contacts, visit
www.sartorius.com/octet-support

Specifications subject to change without notice.
Copyright Sartorius Lab Instruments GmbH & Co. KG.
For Research Use Only.
AN-4014 Rev D
Status: 11 | 2022
